# Equitable Open Access Publishing: Changing the Financial Power Dynamics in Academia

**DOI:** 10.9745/GHSP-D-21-00145

**Published:** 2021-12-31

**Authors:** Dominique Vervoort, Xiya Ma, Hloni Bookholane

**Affiliations:** a Institute of Health Policy, Management and Evaluation, University of Toronto, Toronto, Ontario, Canada.; b Faculty of Medicine, Université de Montréal, Montréal, Québec, Canada.; c Department of Medicine, Groote Schuur Hospital, University of Cape Town, Cape Town, South Africa.

## Abstract

The growth in open access publishing in academia benefits readership but disproportionally hinders unfunded or lesser-funded researchers. Few journals create comprehensive means to bridge these inequities, calling for a shift in academic publishing practices.

## Key Messages

- Open access publishing is steadily growing but associated with high article processing charges that exacerbate disparities between funded and unfunded researchers.
- Early-career and underrepresented researchers often are not eligible for waivers or discounts, thus resulting in either publishing barriers or financial hardship.
- Journals should adopt equitable solutions that enable every author to pursue open access publishing regardless of one's funding status or affiliation.
- Publishing companies should rethink open access publishing models to reduce the financial barriers for readers and authors alike.

Open access (OA) publishing is increasing, allowing articles to be read by anyone, anywhere. The publishing costs for these articles (article processing charges, APCs) are typically paid by the authors or their respective funders.^1^ In global health, authors pay an average of US$2,732 per OA publication.^2^ Articles that are freely accessible are more read, shared, and cited, ultimately benefitting scientific discourse and integration in public health, medicine, and other sciences.^1^ In response to the growing interest in OA publishing, journals are increasingly adopting OA models: some adopt hybrid models that allow authors to choose whether or not to publish OA, some adopt full OA, and some simply create an entirely new sister journal as an OA alternative to their own. However, few create means to support authors who are not funded by research grants, their institutions, or institutional agreements.^3^^,^^4^ Those that do should be commended for taking this step, especially given how rare such genuinely equitable OA models are.

To date, a majority of journals remain hybrid, allowing both subscription-based publishing (i.e., not OA, no APCs to publish) and an OA option (i.e., freely accessible for readers, but APCs to publish). For example, in the fields of cardiology and cardiac surgery, 60.9% of journals are hybrid.^4^ Although this may appear a sensible approach, hybrid journals rarely provide waivers or considerable discounts to authors who cannot afford such APCs, which typically range from a few thousand US dollars to a staggering US$11,000.^5^ In addition, hybrid journals have median APCs of up to 50% higher compared to fully OA journals, as observed in cardiovascular journals (median US$3,250 (interquartile range, IQR: US$3,000–US$3,500) for hybrid journals vs. a median US$2,100 (IQR: US$1,404–US$2,538) for fully OA journals).^4^ Some hybrid journals consider requests for waivers on a case-by-case basis; this is commonly decided based on the first or corresponding author's country affiliation. Authors from low- or middle-income countries (LMICs) and/or with affiliations in LMICs deservedly tend to be favored, although they are not always successful in obtaining a waiver or discount. However, few hybrid journals consider requests from authors who are not from LMICs but who may not be able to afford APCs (e.g., graduate students, researchers without grant or institutional funding, underrepresented minorities), referring them to the option to publish under the subscription model for free. At large, only 37.4% of hybrid cardiovascular journals provided any form of waiver or discount.^4^ Along the same lines, fully OA journals do not provide a subscription-only alternative, completely sidelining those unable to afford APCs and unable to obtain a waiver.

While the intention of increasing access to quality research from LMICs is laudable, few journals process waivers automatically, commonly requiring researchers to submit extensive applications and not always be provided with a full waiver. Moreover, many journals also exclude authors from upper-middle-income countries, such as Brazil and South Africa, from the waiver or discount option and require them to fully cover the APCs. This occurs despite substantial financial barriers that researchers from upper-middle-income countries experience because of great variation in the ability to obtain institutional funding or pay out-of-pocket.^4^ These reasons may partly explain why researchers from LMICs are more likely to cite journals with lower APCs, whereas researchers from high-income countries (HICs) are more likely to cite journals with higher APCs.^2^ One explanation of this phenomenon would be that LMIC researchers access and publish their work in OA journals that are financially more attainable to them, whereas this barrier is rarely an issue for HIC researchers. This results in silos that decrease LMIC research visibility to a wider audience. Approximately 94% of APCs are paid to journals owned by the 10 largest publishers from HICs, a model that sustains an oligopoly that prevents publishers from feeling the need to reduce APCs, even if making a profit.^2^ The current system further does not consider inequity within HICs that leads to the authors' inability to afford APCs for such fully OA journals. This limits the dissemination of information, which can lead to significant consequences for policy makers who are best placed to address systemic issues driving public health disparities.

While the intention of increasing access to quality research from LMICs is laudable, few journals process waivers to authors automatically.

There will always be costs to publish quality research due to journals' fixed and variable expenses. Journals have salaried editorial staff, who manage journals' administrative processes, proofread submissions, check for plagiarism, and send submissions for peer review. After an article is accepted, publishing companies or outsourced companies ensure formatting and typesetting for publication. Further, journals have costs to host and manage their online content, website, marketing, promotion, advertising, indexing, rights, and more. Lastly, the website, submission system, and files require a secure server to host a journal. These costs can be considerable, as they depend on contextual factors (e.g., salaries tied to standard of living, outsourcing tied to company size, reputation, and services, and hardware or software tied to suppliers and markets).

However, APCs by some journals may be higher than the costs to publish. Editorial boards and peer reviewers are typically not paid and contribute their voluntary time to much of the publishing process. Further, journals were traditionally printed; today, journals have online formats with many journals moving away from print versions due to the costs and ecological impact. OA articles are exclusively digital, avoiding printing costs. Lastly, most journals are either partially subsidized (e.g., by societies or institutions) or have sponsored partnerships that cover a considerable portion of journals' fixed costs. Some indexed journals have shown that APCs need not be high: compared to thousands of dollars in most journals, APCs are US$1,749 for PLoS One, US$399 for PeerJ, and free for Cureus.^6^ Although journals and publishers rarely report or even know the true costs per article, they have been reported as low as US$290–US$300 per article with some publishers, questioning the added value of high APCs.^6^

The coronavirus disease (COVID-19) pandemic has greatly illustrated the power of OA and open science, as publishers and journals decided to make COVID-19-related research freely accessible to all. This crisis could be an opportunity to rethink the business models of scientific publication and empower different stakeholders to sustain this practice beyond the pandemic. For instance, inspiration could be drawn from local journals in Latin America that have long embodied such OA practices as a result of their “widespread ethos of free-to-publish and free-to-read research," by which they often even forgo APCs altogether.^7^ Public health, by its very nature, should pursue exactly that: evidence-based information available to all, not just for those able to afford journal access or fortunate enough to have the right academic affiliation. As an example, primary care professionals, community health workers, and nongovernmental organizations are at the front lines of global and public health but are rarely able to freely access scientific literature; ironically, as they often contribute significantly to performing the research that is done. What ethical argument prevents them from accessing materials published by the billion-dollar industry that is academic publishing, whose profit margins are as high as 20%–30%?^8^

The COVID-19 pandemic could be an opportunity to rethink the business models of scientific publication and empower different stakeholders to sustain this practice.

Recent trends and transformative agreements in the publishing landscape do provide hope for more equitable publishing practices in the near future. First, European institutions, along with major funders such as the Wellcome Trust and Bill & Melinda Gates Foundation, have signed on to the adoption of “Plan S,” which requires articles supported by public grants to be published in OA as of 2021. Although this greatly benefits European researchers, it may place increasing barriers for those from other institutions unless similar models are adopted elsewhere.^9^ Nevertheless, many concerns, including those of few journals following Plan S recommendations, are slowly being addressed as journals increasingly sign on^10^ and flexibility of journal choices is expanded in response to the academic community's requests.^11^ Second, Research4Life is an initiative in collaboration with the World Health Organization, other United Nations agencies, Cornell University, Yale University, the International Association of Scientific, Technical, and Medical Publishers, and nearly 200 publishers. Through the Hinari Access to Research for Health Programme, Research4Life provides free or low-cost access to health-related academic literature for researchers and institutions in LMICs. However, given the current limited scope of the Hinari program, increased international support from all parties is required.^12^ Lastly, read-and-publish agreements, such as those supported by the European Public Health Association, have been adopted in various countries.^3^ This requires countries or consortiums to pay publishers a lump sum to access articles, which is used for publishing costs, thereby creating a theoretical cost-neutral model that ensures OA for readers and authors from these countries or institutions.

Implementation of novel and more equitable OA models and practices will be critical. Barriers to publishing are widespread for researchers worldwide as research grants are minimal and highly concentrated in select countries and institutions and obtaining them has only become more challenging during the COVID-19 pandemic.^8^ Moreover, the inaccessibility of both OA and subscription journals is giving rise to more predatory journals, which promise quick and OA publication of articles with minimal to no review against a fee that is substantially lower than average APCs. This option is becoming increasingly attractive to vulnerable authors worldwide and has only been amplified by the publish-or-perish culture in academia, the notable barriers to scientific publication, and the COVID-19 pandemic.^13^ Although these predatory journals are OA, they pose a threat to the access of evidence-based information as they typically publish misinformation, do not send articles for peer review, and keep research published in these journals hidden from the scientific community because they are generally not indexed in established databases, all of which may negatively affect authors' reputations.^13^ Thus, reducing the barriers to scientific publishing, especially regarding the high APCs that impede unfunded researchers from pursuing fully OA journals and often even hybrid journals, is an important step toward equity in today's academic environment.

Various opportunities arise. Journals and publishers should become more transparent about their use of funds to justify high APCs, especially when non-APC revenue is clearly generated (e.g., advertisements). Further, journals not associated with large publishers can offset fixed costs by collaborating with institutions, agencies, or societies to share servers and receive subsidies. Similarly, strategic partnerships with sponsors can generate revenue for fixed costs. Lastly, journals and publishers ought to consider tiered fee discounts and waivers—where possible, automated—to allow lesser-funded or unfunded researchers to pursue OA. Given the profit margins observed among large publishers, these waivers and discounts can be offset accordingly and be considered an investment in the future of academic publishing and accelerate medicine and public health.

Journals and publishers should become more transparent about their use of funds to justify high APCs.

Journals adopting OA models are to be commended but should be encouraged to increase opportunities to reduce publication fees and support unfunded or lesser-funded authors. Open access publishing is not only the future; it is the key to regaining public trust in science, retaining early-career academics, strengthening public and health policy, addressing public health disparities, and leveling the playing field for all researchers alike.

## Notes

**Peer Reviewed**

**First published online:** December 9, 2021.
